# Public Knowledge, Beliefs and Behavior on Antibiotic Use and Self-Medication in Lithuania

**DOI:** 10.3390/ijerph120607002

**Published:** 2015-06-17

**Authors:** Eglė Pavydė, Vincentas Veikutis, Asta Mačiulienė, Vytautas Mačiulis, Kęstutis Petrikonis, Edgaras Stankevičius

**Affiliations:** 1 Institute of Physiology and Pharmacology, Lithuanian University of Health Sciences, A. Mickeviciaus Str. 9, Kaunas 44307, Lithuania; E-Mail: edgaras.stankevicius@lsmuni.lt; 2 Institute of Microbiology and Virology, Lithuanian University of Health Sciences, Kaunas 49264, Lithuania; E-Mail: vincentas.veikutis@med.kmu.lt; 3 Department of Anesthesiology, Lithuanian University of Health Sciences, Kaunas 50161, Lithuania; E-Mails: asta.svitojute@gmail.com (A.S.); vt.maciulis@gmail.com (V.M.); 4 Department of Neurology, Lithuanian University of Health Sciences, Kaunas 50161, Lithuania; E-Mail: kestutispetrikonis@yahoo.com

**Keywords:** antibiotics, knowledge, rational antibiotic use, self-medication, risk factors, antibiotic resistance, parent behavior, Lithuania

## Abstract

Irrational antibiotic use has led society to antibiotic resistance—a serious health problem worldwide. This study aimed to assess public knowledge, beliefs, and behavior concerning antibiotic use and self-medication in Lithuania. The cross-sectional survey method was processed using a validated questionnaire in different regions of Lithuania. In total, 1005 adults completed the questionnaire and were included in the study. More than half of the respondents (61.1%) had poor knowledge of antibiotics. Almost half of the respondents incorrectly identified antibiotics as being effective either against viral (26.0%) or mixed (bacterial and viral) infections (21.7%). The respondents with lower educational qualifications (OR = 2.515; 95% CI 1.464–4.319; *p* = 0.001) and those from rural areas (OR = 1.765; 95% CI 1.041–2.991; *p* = 0.035) were significantly less knowledgeable of antibiotics. There was no significant difference between genders, different age groups, or different parenthood status. The determined level of self–medication with antibiotics was 31.0%. The men (OR = 1.650; 95% CI 1.120–2.430; *p* = 0.011), the respondents from rural areas (OR = 2.002; 95% CI 1.343–2.985; *p* = 0.001), and those without children (OR = 2.428; 95% CI 1.477–3.991; *p* < 0.001) were more likely to use antibiotics in self-medication. Lithuanian residents’ knowledge of antibiotics is insufficient. More information about antibiotic use should be provided by physicians and pharmacists. Self-medication with antibiotics is a serious problem in Lithuania and requires considerable attention.

## 1. Introduction

Irrational antibiotic use has led society to antibiotic resistance—a serious health problem worldwide, which is now trying to be solved by many various approaches. In 2011, the World Health Day theme was “Combat drug resistance: no action today means no cure tomorrow” and for this occasion the World Health Organization introduced a six-point policy package to fight against the spread of antimicrobial resistance [1]. This reflects the importance of the problem and the need to undertake some serious actions in all population groups, involved in the growth of antimicrobial resistance and irrational antibiotic use. 

There are various factors which may influence an increase in irrational antibiotic use. Many studies have reported that antibiotic regime non-adherence and inappropriate antibiotic use are strongly associated with public awareness and knowledge of antibiotics [2,3,4,5]. Factors associated with public knowledge of antibiotics have been reported to be demographic characteristics, including gender [2,4,6,7,8], age [6,8,9,10,11,12], race [7,9], education level [3,4,6,7,8,9,10,12,13,14,15], family income [4,8,14], place of residence [13,14], as well as other factors, such as lack of advice regarding rational antibiotic use, given by a physician [16]. A comparative European study has shown that respondents from Lithuania were significantly less knowledgeable of antibiotics, compared with such countries as Sweden, Belgium, Austria, and the Netherlands [17].

Another important issue related to the increase in antibiotic resistance is self-medication, which is defined as the acquisition of antibiotics and self-administering them (or administering them to children) with the aim of treating perceived infection [18]. There are clear differences between the prevalence rates of self-medication with antibiotics among different European countries, ranging from 5% to 45% [3,11,19,20,21,22,23,24,25] in the general population. A previous study processed in Lithuania showed that prevalence of self-medication with antibiotics was 22% [25]. It should be noted that there is an easy access to health care services in Lithuania. There is a high number of physicians, general practitioners, and pharmacists, favorably comparable with numbers in more advanced countries. In 2012, there were 42.4 physicians, 6.3 general practitioners, and 10.3 pharmacists per 10,000 inhabitants, as well as 8.0 visits to physicians in general per one inhabitant per year. In Lithuania, antibiotics are prescribed and treatment can be continued by a physician only.

Irrational antibiotic use reflects not only patients’ failure to comply with physician’s instructions on how to use antibiotics adequately, but is also associated with inappropriate antibiotic prescribing. Rational antibiotic therapy should be based on the correct indication, the right drug and dosage, the drug of the first choice, the appropriate period of use, and the lowest treatment costs [26]. All antibiotic prescription events that do not comply with these conditions should be considered as irrational prescribing. A study processed in Lithuania determined that, only in 9.5% of all cases, antibiotics were administered in keeping with the recommendations for rational antibiotic therapy; according to indications, appropriate antibiotics were prescribed in 42.9% of cases [27]. This shows that not only patients but also physicians must be considered as a key group causing the increase in antibiotic resistance. 

Due to wide cross-national differences in antibiotic use and public knowledge of antibiotics, determination of key populations requiring educational interventions is needed in each country prior to the establishment of effective educational programs. Studies regarding public knowledge of antibiotics and self-medication with antibiotics are rare [17,25]. Our study was aimed at determining which Lithuanian society groups were more likely to use antibiotics in self-medication. The main purpose of this study was to assess public knowledge, beliefs, and behavior concerning antibiotic use and self-medication in Lithuania.

## 2. Methods 

### 2.1. Study Design 

A prospective cross-sectional study was designed based on a validated anonymous self-administered questionnaire. Ethical approval was obtained from the Lithuanian University of Health Sciences Bioethics Centre. To be eligible for this study, participants had to provide signed informed consent. The anonymous survey was processed in 16 community pharmacies located in 4 different regions of Lithuania, covering the whole country. All the selected pharmacies were similar in terms of size, location (near hospitals, family health centers, or physician offices), and patient load per month. Respondents were recruited by our research team. All the patients who came to the selected community pharmacies during the study period were asked to fill out the questionnaire at the pharmacy, regardless of antibiotic acquisition at the time of visit or antibiotic use at any time in life. Respondents under 18 and those with occupation related to health care were not included in this study. A total of 1,005 respondents were eligible for the final analysis. 

### 2.2. Study Instrument

The anonymous survey method was performed using the original validated 37-item questionnaire (in Lithuanian) containing both open- and close-ended (multiple-choice) questions. The questionnaire was developed by our research group based on a previously conducted literature review [4,7,9,17,18,20,23,24,28] and specific cultural considerations. The validity and reliability of the questionnaire were evaluated in a pilot study, in a sub-sample of 30 participants, to ensure that the questionnaire would be appropriate, comprehensive, and understandable among prospective respondents. The pilot testing allowed quality improvement of several questions by wording modification and achieved high internal consistency and reliability (Cronbach’s α = 0.75). A total of 37 questions were included in the final instrument, which was divided to five parts:

#### 2.2.1. Part 1: Demographic Characteristics

There were six questions in Part 1 regarding the demographic characteristics of the study population, including gender, age, level of education, occupation, place of residence, and parenthood status. Urban place of residence was defined as living in a city or town with the population size of more than 3000 inhabitants (according to Lithuanian territorial administrative units). Parenthood status was defined as having children under 18. The data regarding occupation were only used to exclude respondents with the occupation related to health care. 

#### 2.2.2. Part 2: Antibiotic Use and Self-Evaluation of Antibiotic Knowledge

Part 2 consisted of seven questions. The respondents were asked to indicate prior antibiotic use at any time in life and the approximate frequency of antibiotic use (if applicable). The antibiotic users were also asked about their most recent antibiotic use (reason, for which they used antibiotics and antibiotic name used for the therapy; both open-ended questions). They were also asked to indicate the main sources of information regarding antibiotic use. Before filling out Part 3, the respondents had to evaluate themselves and to assess their knowledge of antibiotics using a 6-point numerical rating scale (0–5), where the end points were the extremes of no knowledge and excellent knowledge. According to their self-evaluation, we divided the respondents into three groups as follows: 4–5 points—good knowledge, 2–3 points—average knowledge, and 0–1 point—poor knowledge of antibiotics. The results of self-evaluation were later compared with the actual knowledge of antibiotics.

#### 2.2.3. Part 3: Knowledge and Beliefs Concerning Antibiotic Use

There were nine questions in Part 3 regarding knowledge and beliefs related to antibiotic use. We selected five questions from this part to assess respondents’ actual knowledge of antibiotics. The questions inquired information about rational antibiotic use, antibiotic effectiveness against bacterial and/or viral infections, indications for antibiotic use, and the appropriate period of antibiotic use. The number of correct answers to these questions (0–5) indicated the actual knowledge score as follows: 4–5 points—good knowledge, 2–3 points—average knowledge, and 0–1 point—poor knowledge of antibiotics. Part 3 also included the questions regarding respondents’ beliefs on safety of antibiotic use and possible adverse drug reactions (ADRs). 

#### 2.2.4. Part 4: Behavior regarding Antibiotic Use and Self-Medication with Antibiotics

Part 4 consisted of eight questions related to antibiotic use behavior, antibiotic acquisition, and self-medication with antibiotics. Self-medication prevalence was measured among the antibiotic users. Self-medication was determined based on the answers to questions in Part 4 and considered as the selection and use of antibiotics by the study participants (or their family members) to treat self-recognized or self-diagnosed condition at any time in life. 

#### 2.2.5. Part 5: Parents’ Behavior Concerning Antibiotic Administration to Children

The last part of the questionnaire was designed for parents and included seven questions. The parents were asked about their children’s most recent antibiotic use if applicable (reason, for which they used antibiotics and antibiotic name used for the therapy; both open-ended questions). Other questions in Part 5 assessed how the parents supervised their children’s health problems related to antibiotic use.

### 2.3. Sample Size

A sample size calculation was performed using the Raosoft sample size calculator [29] with a 5% margin of error, a 95% confidence level, the population size of 3003.6 k (population size of Lithuania, according to the data of the Statistics Lithuania), and a 50% response distribution. The calculation resulted in a sample size of 385. As the study was processed in community pharmacies, to increase reliability of sampling and sampling-based generalizability, the required sample size was doubled. With the expected response rate of 50%–60%, a total of 1300 questionnaires were distributed to the selected pharmacies.

### 2.4. Statistical Data Analysis

A descriptive and comparative statistical data analysis was processed with the SPSS 17.0 (SPSS Inc., Chicago, IL, USA). Frequencies and cross tables of preselected variables were calculated and χ^2^ tests were performed to identify variables associated with dependent variables—knowledge of antibiotics and self-medication. Risk factors of poor antibiotic knowledge (among all the respondents) and self-medication (among the antibiotic users) was first evaluated using bivariate logistic regression. Variables with *p* < 0.25 in the bivariate analysis were included in the multivariate logistic regression analysis to determine the factors that are independently associated with each dependent variable. Only the results of multivariate logistic regression analysis are reported. Odds ratios (OR), 95% confidence intervals (CI), and *p*-values were calculated for each independent variable. A *p*-value less than 0.05 was considered to be statistically significant.

## 3. Results

A total of 1005 out of 1300 administered questionnaires were returned fully completed and met the inclusion criteria, giving a response rate of 77.3%. Less than half of the respondents (42.1%) were men. The mean age of the respondents was 38.6 ± 13.9 (median 37; range 18–79). Most respondents were from urban places of residence (74.3%) and had university or college education (70.3%). Study population characteristics compared with the Lithuanian general population are detailed in Table 1. 

Nearly all the respondents (93.7%) claimed that they had used antibiotics at some point in their life. One-tenth (9.8%) indicated that they had used antibiotics at least once in 6 months, and one-quarter (24.9%) at least once a year. The main sources of antibiotic information were physicians (64.5%), pharmacists (45.0%), family members or friends (41.8%), and the Internet (40.5%) (study participants could choose more than one possible source of information). Even though physicians were reported to be the main source of information related to antibiotic use by most respondents, one-third of them (34.0%) stated that they got too little or no information about antibiotics from their physician.

The antibiotic users had to identify health problems and antibiotics used for the most recent antibiotic therapy. Almost one-third of the respondents (29.7%) did not indicate the disease or the antibiotic (most of the answers in these cases were “I don’t know” or “I don’t remember”); 18.6% of the respondents indicated that they had used antibiotics for various noninfectious diseases; 5.3% of the respondents confused antibiotics with other medications such as nonsteroidal anti-inflammatory drugs or antipyretics, which are usually used for the symptomatic treatment of common colds. 

**Table 1:** Demographic characteristics of respondents compared with the Lithuanian general population.

Respondents’ Characteristics		n (%)		Lithuania n (%)		
Gender	Male	423	(42.1)	1383.5	k	(46.1)
	Female	582	(57.9)	1620.1	k	(53.9)
Age	18–29	334	(33.2)	496.7	k	(20.3)
	30–39	261	(26.0)	376.3	k	(15.4)
	40–49	204	(20.3)	439.5	k	(18.0)
	50–59	132	(13.1)	423.7	k	(17.3)
	≥60	74	(7.4)	711.1	k	(29.0)
Place of residence	Urban	747	(74.3)	2005.6	k	(66.8)
	Rural	258	(25.7)	998.0	k	(33.2)
Level of education	Lower than university or college	298	(29.7)	1897.1	k	(74.4)
	University or college	707	(70.3)	653.6	k	(25.6)
Parenthood	Yes	621	(61.8)	N/A		
	No	384	(38.2)	N/A		

Before answering the questions related to antibiotic use, the respondents evaluated their knowledge of antibiotics; 22.8% of the respondents rated their antibiotic knowledge as good, 43.8% as average, and 33.4% as poor (Table 2). Poor knowledge of antibiotics was found to be significantly associated with the overestimation of self-knowledge of antibiotics (*p* < 0.001). Though almost half of the respondents (42.0%) evaluated their knowledge of antibiotics accurately, a similar proportion (44.6%) overestimated their knowledge. Moreover, better self-evaluation was associated with higher prevalence of self-medication (*p* < 0.001).

**Table 2:** Self-evaluation of antibiotic knowledge *versus* actual knowledge

Respondents’ Self-Evaluation		Evaluation of Actual Antibiotic Knowledge n (%)										
		Good (4–5 Points)			Average (2–3 Points)			Poor (0–1 Point)			Total	
**Self-evaluation**	Good	39	(3.9)	*	84	(8.4)	**	106	(10.5)	**	229	(22.8)
	Average	49	(4.9)		133	(13.2)	*	258	(25.7)	**	440	(43.8)
	Poor	16	(1.6)		70	(7.0)		250	(24.9)	*	336	(33.4)
	Total	104	(10.3)		287	(28.6)		614	(61.1)			

Notes: * Respondents who evaluated their antibiotic knowledge accurately. ** Respondents who overestimated their knowledge of antibiotics.

According to our scoring of respondents’ knowledge of antibiotics, only one-third of them had good (10.3%) or average (28.6%) knowledge. Almost half of the respondents incorrectly identified antibiotics as being effective either in treating viral (26.0%) or mixed (bacterial and viral) infections (21.7%); 41.7% noted common cold as an appropriate indication for antibiotic use. A considerably low percentage of the respondents (15.1%) indicated 3 days or less to be an appropriate duration of antibiotic use. The majority of the respondents (92.9%) knew that antibiotics could cause ADRs, and most of them were aware of such ADRs as allergic reactions (62.3%), dysbacteriosis (45.7%), nausea and/or vomiting (41.4%), diarrhea (24.4%), and headache (25.0%). The respondents’ knowledge and beliefs of antibiotics are shown in Table 3.

**Table 3:** Respondents’ knowledge and beliefs of antibiotics.

Knowledge and Beliefs	n (%)	
Antibiotics are effective in treating bacterial but not viral infections	526	(52.3)
Antibiotics are effective in treating viral but not bacterial infections	261	(26.0)
Antibiotics are effective in treating both bacterial and viral infections	218	(21.7)
Appropriate indications for antibiotic use *		
Inflammation	563	(56.0)
Pneumonia	690	(68.7)
Common cold	419	(41.7)
Fever	95	(9.5)
Sore throat	28	(2.8)
Cough	11	(1.1)
Appropriate duration of antibiotic use		
≤3 days	152	(15.1)
4–6 days	427	(42.5)
≥7 days	426	(42.4)
Appropriate period of antibiotic use *		
Period indicated by physician or pharmacist	821	(81.7)
Period indicated in drug leaflet	320	(31.8)
Antibiotics should be used until disappearance of symptoms	135	(13.4)
Antibiotics should be used until relief of symptoms	102	(10.1)
Period indicated by family members or friends	43	(4.3)
Antibiotics are safe medications		
Yes	255	(25.4)
No	470	(46.8)
I don't know	280	(27.9)
Antibiotics can cause adverse drug reactions		
Yes	934	(92.9)
No	14	(1.4)
I don’t know	57	(5.7)

Notes: Frequency and percentage of respondents agreeing on statements; * Respondents could agree on more than one statement.

Table 4 shows the multivariate logistic regression analysis of factors independently associated with poor knowledge of antibiotics and self-medication. We found two factors independently associated with poor knowledge of antibiotics including education level and place of residence (Table 4). The respondents with lower than university or college education (OR = 2.515; 95% CI 1.464–4.319; *p* = 0.001) and the respondents from rural places of residence (OR = 1.765; 95% CI 1.041–2.991; *p* = 0.035) were significantly less knowledgeable of antibiotics. There was no significant difference between men and women, different age groups, or respondents with a different parenthood status.

**Table 4:** Multivariate logistic regression analysis of factors independently associated with poor knowledge of antibiotics and self-medication.

Factor	Categories	OR	95% CI for OR	*p*
**Poor antibiotic knowledge (n = 1005)**				
Gender	Male	1.225	0.776–1.933	0.384
	Female	1.000		
Age	18–29	2.038	0.845–4.916	0.113
	30–39	1.341	0.604–2.975	0.471
	40–49	1.077	0.474–2.449	0.859
	50–59	2.283	0.857–6.077	0.099
	≥60	1.000		
Education	University or college	1.000		
	Lower than university or college	2.515	1.464–4.319	**0.001**
Place of residence	Urban	1.000		
	Rural	1.765	1.041–2.991	**0.035**
Parenthood	Yes	1.000		
	No	1.514	0.836–2.744	0.171
**Self-medication (n = 942)**				
Gender	Male	1.650	1.120–2.430	**0.011**
	Female	1.000		
Age	18–29	0.299	0.146–0.611	**0.001**
	30–39	0.469	0.236–0.930	**0.030**
	40–49	0.729	0.358–1.484	0.384
	50–59	0.890	0.431–1.840	0.754
	≥60	1.000		
Education	University or college	1.000		
	Lower than university or college	0.719	0.463–1.116	0.142
Place of residence	Urban	1.000		
	Rural	2.002	1.343–2.985	**0.001**
Parenthood	Yes	1.000		
	No	2.428	1.477–3.991	**<0.001**
Knowledge of antibiotics	Good	1.168	0.631–2.163	0.620
	Average	1.037	0.675–1.593	0.869
	Poor	1.000		

Note: *p* < 0.05 was considered to be statistically significant.

More than one-quarter of the antibiotic users (27.8%) stated that they had bought or used antibiotics without prescription at some point in life. The results showed that the main sources of antibiotic supply without prescription were community pharmacies (72.7%) or leftover of antibiotics stored in the household (14.8%). Supplies by family members and friends accounted for 9.9%. The determined level of self-medication with antibiotics among the study population was 31.0%. The independent risk factors of self-medication were found to be gender, place of residence, and parenthood status (Table 4: Multivariate logistic regression analysis of factors independently associated with poor knowledge of antibiotics and self-medication). The men (OR = 1.650; 95% CI 1.120–2.430; *p* = 0.011), the respondents from rural place of residence (OR = 2.002; 95% CI 1.343–2.985; p = 0.001), and the respondents without children (OR = 2.428; 95% CI 1.477–3.991; *p* < 0.001) were more likely to use antibiotics in self-medication. Higher education level or better knowledge of antibiotics were not associated with the increased risk of self-medication. Interestingly, the younger respondents (age group 18–29: OR = 0.299; 95% CI 0.146–0.611; *p* = 0.001; age group 30–39: OR = 0.469; 95% CI 0.236–0.930; *p* = 0.030) were less likely to use antibiotics in self-medication. 

Out of 621 respondents who had children under 18, almost all (92.8%) stated that their children had used antibiotics at some point in life. More than one-tenth of the respondents (12.3%) indicated that their children had used antibiotics at least once in 6 months, and almost half of them (43.2%)—at least once a year. Almost half of the parents (41.3%) did not indicate the disease or the antibiotic that was administrated to their children during the most recent antibiotic therapy; 14.4% of the respondents indicated that their children had used antibiotics for noninfectious diseases and only 1.4% of respondents confused antibiotics with other medications. Most parents noted supervising the use of antibiotics according to the instructions given by a physician (58.0%) or a pharmacist (23.1%). More than one-tenth of the parents reported administering antibiotics to children according to their own knowledge (8.5%) or advice given by family members and friends (5.6%); 4.8% parents stated that they would never give antibiotics to their children.

## 4. Discussion

This study revealed important findings, related to inadequate public knowledge of antibiotics. Our results showed that antibiotic knowledge among Lithuanian population was insufficient as almost two-thirds of the respondents (61.1%) had poor knowledge of antibiotics. A large proportion of the respondents (41.7%) thought that antibiotics worked on common cold, 26.0% that antibiotics were effective in treating viral infections, and 21.7% that antibiotics were effective in treating mixed (bacterial and viral) infections. Moreover, people tended to overestimate their knowledge of antibiotics, which might lead to increased non-adherence and self-medication. The respondents with lower educational qualifications and those from rural places of residence were less knowledgeable about antibiotics. Self-medication behavior was identified in almost one-third of the antibiotic users (31.0%) and was found to be significantly associated with male gender, rural place of residence, and absence of children. 

Insufficient public knowledge of antibiotics has been previously reported in various countries and regions [3,11,15,17,19,30]. Our study revealed two main factors significantly associated with knowledge of antibiotics and such findings are comparable with previously published data. These factors were level of education [3,4,6,7,8,9,10,12,13,14,15] and place of residence [13,14]. The respondents with high education level and those living in urban areas were more aware of antibiotics and their rational use. However, we did not find any significant differences between genders [2,4,6,7,8] or different age groups [6,8,9,10,11,12], as reported elsewhere.

Our study showed pleasing results that health care professionals were the main source of antibiotic information. As physicians and pharmacists play an important role in contributing to public knowledge of antibiotics, they must be considered as a powerful instrument for the increase in rational antibiotic use in society and the improvement of patients’ behavior regarding antibiotic use. Both, physicians and pharmacists must be encouraged by national health programs to provide more information about appropriate antibiotic use and its’ importance while prescribing or dispensing these medications. Moreover, all academic institutions should pay huge attention to education of future health care professionals, providing them good rational antibiotic therapy knowledge and effective patient consultation skills [31,32,33,34,35]. 

One of the most important and novel findings in our study is the relationship between self-evaluation of antibiotic knowledge and the actual knowledge of antibiotics. Interestingly, the respondents with poor knowledge of antibiotics tended to overestimate their knowledge. Such overestimation and confidence in self-knowledge was found to negatively affect the prevalence of self-medication behavior. We were not able to find any previous surveys investigating respondents’ opinion on their knowledge of antibiotics and requiring study participants to perform self-assessment. This finding seems to be very substantial, demonstrating the need for broad educational interventions. Various studies have presented local, national, and regional educational programs and public campaigns, processed to increase antibiotic awareness with various success rates [35,36,37]. This tool should be considered by health care authorities in order to improve rational antibiotic use and decrease self-medication rates in Lithuania. Major attention should be focused on the rural public, as both the rate of poor knowledge of antibiotics and the prevalence of self-medication were identified to be significantly higher in this part of the population. 

Our study results show that there is still an easy access to antibiotics without supervision of a physician, as 27.8% of the respondents stated that they had bought or used antibiotics without prescription, despite the national regulation that strictly defines antibiotics as prescription-only medicines. The main source of such antibiotic supply was a community pharmacy. This may indicate that the Lithuanian health care system and especially community pharmacies are still struggling to cope with their task in enhancing rational antibiotic use. It should be noted that a similar situation has been reported by many previous studies [18,20,21,23,28,38] and community pharmacies remain the main source of nonprescription antibiotic supply, demonstrating the need for effective tools, enforcing stricter supervision of antibiotic sale.

According to our results, the estimated self-medication with antibiotics in Lithuania remains high (31.0%). In comparison with previously reported data, this level has increased by 10% since 2006 [25]. However, such variance may be due to the differences between study populations and selection bias; therefore, it cannot be entirely comparable. The estimated self-medication level of our study was found to be higher in Lithuania than in such European countries as the United Kingdom (5.0%) [3], Macedonia (17.8%) [19], Portugal (18.3%) [39], and Turkey (19.1%) [24], but lower than in Italy (32.7%) [11], Spain (41.0%) [20], Poland (41.4%) [21], Romania (44.0%) [22], and Greece (44.6%) [23]. This variance is sufficiently comparable to the differences in the outpatient antibiotic consumption among these countries. Antibiotic consumption (expressed in defined daily doses per 1,000 inhabitants per day; DID) in Lithuania (19.72) was found to be lower than in Italy (28.66), Poland (23.59), Portugal (22.94), and Greece (38.64), similar as in Spain (19.68), and higher than in the United Kingdom (17.27) [40]. Even though antibiotic consumption was reported to be almost twice lower in Romania (10.19) in comparison with Lithuania, self-medication with antibiotics was even 13% lower in Lithuania. As mentioned, all Lithuanian citizens and registered long-term residents have an easy access to free state-funded health care services. Therefore, issues in the health care system or no access to a physician’s consultation should not be considered as a factor increasing prevalence of self-medication. 

The results of our study revealed that self-medication with antibiotics in Lithuania was significantly higher in the men, the respondents from rural places of residence, and the respondents without children. Higher prevalence of self-medication with antibiotics in men has been reported by several previous studies [24,38,39]. However, most researchers found no differences between men and women [10,11,19,20,28] or even considered female gender as a risk factor of self-medication with antibiotics [3,18]. In our study, risk of antibiotic use in self-medication was found to be significantly lower among the respondents under 40 years of age. Differences between the prevalence rate of self-medication in different age groups have been reported in the majority of previous studies. However, most of these studies identified younger age to be significantly associated with the increased antibiotic use in self-medication [11,16,18,29,32] and only a few studies indicated middle age as a risk factor of self-medication [18,39]. Our study was not able to support consistent associations between self-medication and such variables as education level and knowledge of antibiotics. 

Study participants demonstrated sufficiently appropriate behavior regarding supervision of antibiotic use of their children. Most parents followed the instructions given by a health care professional (physician or pharmacist), while only a small percentage of parents reported self-administering antibiotics to their children or trusting family members’ and friends’ advice. These results demonstrate that parents take their children’s health problems more seriously and responsibly than their own. 

## 5. Strengths and Limitations 

In our study, we obtained a high response rate (77.3%), which minimizes potential response bias because a response rate greater than 50%–60% is considered to be sufficient [41]. To date, this is the largest study on public knowledge of antibiotics in Lithuania. Knowledge of antibiotics as well as self-medication with these medications among the Lithuanian population has only been studied to a limited extent [17,25]. Other strengths of this study include all desired age groups, high as well as low level of education, and respondents from both urban and rural places of residence. Most of the study population characteristics are reasonably comparable with those of the general Lithuanian population.

In spite of the large sample size and high response rate, several limitations of this study should be noted. As the study was based on a self-administered questionnaire, the data were presented from recollection of respondents’ memory, and, thus, in some cases it was subjective. The distribution of the respondents with high and low education level was not even. We obtained a small percentage of the respondents over 60 years old. Other limitations include education level and previous use of antibiotics being self-reported. Family income, employment status, number of children, marital status, and health status were not analyzed as covariates possibly associated with knowledge of antibiotics and self-medication, although several previous studies have suggested this association [11,18,24,28,38].

## 6. Conclusions 

In conclusion, this study revealed a high percentage of inappropriate antibiotic knowledge and a high rate of self-medication with antibiotics among Lithuanian population. Education programs should be developed, targeting specific public groups identified in this study, with lower antibiotic knowledge and higher self-medication risk. Finally, the attention of health care policy makers should be focused on physicians and pharmacists, as the main information providers of rational antibiotic use, as well as on community pharmacies, identified as the main source of nonprescription antibiotics.

## References

[1] WHO World Health Day 2011: Policy Briefs. http://www.who.int/world-health-day/2011/policybriefs/en/.

[2] Chan Y.H., Fan M.M., Fok C.M., Lok Z.L., Ni M., Sin C.F., Wong K.K., Wong S.M., Yeung R., Yeung T.T., Chow W.C., Lam T.H., Schooling C.M. (2012). Antibiotics nonadherence and knowledge in a community with the world’s leading prevalence of antibiotics resistance: Implications for public health intervention. Amer. J. Infect. Control.

[3] McNulty C.A., Boyle P., Nichols T., Clappison P., Davey P. (2007). Don’t wear me out—The public’s knowledge of and attitudes to antibiotic use. J. Antimicrob. Chemother..

[4] You J.H.S., Yau B., Choi K.C., Chau C.T.S., Huang Q.R., Lee S.S. (2008). Public knowledge, attitudes and behavior on antibiotic use: A telephone survey in Hong Kong. Infection.

[5] Awad A.I., Aboud E.A. (2015). Knowledge, Attitude and practice towards antibiotic use among the public in Kuwait. PLoS ONE.

[6] Hoffmann K., Ristl R., Heschl L., Stelzer D., Maier M. (2014). Antibiotics and their effects: What do patients know and what is their source of information?. Eur. J. Public Health.

[7] Lim K.K., Teh C.C. (2012). A Cross sectional study of public knowledge and attitude towards antibiotics in Putrajaya, Malaysia. South. Med. Rev..

[8] Barah F., Gonçalves V. (2010). Antibiotic use and knowledge in the community in Kalamoon, Syrian Arab Republic: A cross-sectional study. East. Mediterr. Health J..

[9] Ling Oh A., Hassali M.A., Al-Haddad M.S., Syed Sulaiman S.A., Shafie A.A., Awaisu A. (2011). Public knowledge and attitudes towards antibiotic usage: A cross-sectional study among the general public in the state of Penang, Malaysia. J. Infect. Dev. Ctries..

[10] Jose J., Jimmy B., Alsabahi A.G.M.S., Al Sabei G.A. (2013). A study assessing public knowledge, belief and behavior of antibiotic use in an omani population. Oman Med. J..

[11] Napolitano F., Izzo M.T., di Giuseppe G., Angelillo I.F. (2013). Public knowledge, attitudes, and experience regarding the use of antibiotics in Italy. PLoS ONE.

[12] Kim S.S., Moon S., Kim E.J. (2011). Public knowledge and attitudes regarding antibiotic use in South Korea. J. Korean Acad. Nurs..

[13] Godycki-Cwirko M., Cals J.W.L., Francis N., Verheij T., Butler C.C., Goossens H., Zakowska I., Panasiuk L. (2014). Public beliefs on antibiotics and symptoms of respiratory tract infections among rural and urban population in Poland: A questionnaire study. PLoS ONE.

[14] Mouhieddine T.H., Olleik Z., Itani M.M., Kawtharani S., Nassar H., Hassoun R., Houmani Z., Zein Z.E., Fakih R., Mortada I.K., Mohsen Y., Kanafani Z., Tamim H. (2015). Assessing the Lebanese population for their knowledge, attitudes and practices of antibiotic usage. J. Infect. Public Health.

[15] Alzoubi K., Al-Azzam S., Alhusban A., Mukattash T., Al-Zubaidy S., Alomari N., Khader Y. (2013). An audit on the knowledge, beliefs and attitudes about the uses and side-effects of antibiotics among outpatients attending 2 teaching hospitals in Jordan. East. Mediterr. Health.

[16] Fernandes M., Leite A., Basto M., Nobre M.A., Vieira N., Fernandes R., Nogueira P., Nicola P.J. (2014). Non-adherence to antibiotic therapy in patients visiting community pharmacies. Int. J. Clin. Pharm..

[17] Grigoryan L., Burgerhof J.G.M., Degener J.E., Deschepper R., Lundborg C.S., Monnet D.L., Scicluna E.A., Birkin J., Haaijer-Ruskamp F.M., SAR consortium (2007). Attitudes, beliefs and knowledge concerning antibiotic use and self-medication: A comparative European study. Pharmacoepidemiol. Drug Saf..

[18] Awad A., Eltayeb I., Matowe L., Thalib L. (2005). Self-medication with antibiotics and antimalarials in the community of Khartoum State, Sudan. J. Pharm Pharm. Sci..

[19] Ivanovska V., Zdravkovska M., Bosevska G., Angelovska B. (2013). Antibiotics for upper respiratory infections: Public knowledge, beliefs and self-medication in the Republic of Macedonia. Prilozi.

[20] Väänänen M.H., Pietilä K., Airaksinen M. (2006). Self-medication with antibiotics—Does it really happen in Europe?. Health Policy.

[21] Muras M., Krajewski J., Nocun M., Godycki-Cwirko M. (2013). A survey of patient behaviours and beliefs regarding antibiotic self-medication for respiratory tract infections in Poland. Arch. Med. Sci..

[22] Damian L., Lupuşoru C.E., Ghiciuc C.M. (2014). Self-medication with antimicrobial drugs among university students in a northeast region of Romania. Rev. Med. Chir. Soc. Med. Nat. Iasi..

[23] Skliros E., Merkouris P., Papazafiropoulou A., Gikas A., Matzouranis G., Papafragos C., Tsakanikas I., Zarbala I., Vasibosis A., Stamataki P., Sotiropoulos A. (2010). Self-medication with antibiotics in rural population in Greece: A cross-sectional multicenter study. BMC Fam. Pract..

[24] Ilhan M.N., Durukan E., Ilhan S.O., Aksakal F.N., Ozkan S., Bumin M.A. (2009). Self-medication with antibiotics: Questionnaire survey among primary care center attendants. Pharmacoepidemiol. Drug Saf..

[25] Berzanskyte A., Valinteliene R., Haaijer-Ruskamp F.M., Gurevicius R., Grigoryan L. (2006). Self-medication with antibiotics in Lithuania. Int. J. Occup. Med. Environ. Health.

[26] WHO The World Medicines Situation Report. http://who.int/medicines/areas/policy/world_medicines_situation/en/.

[27] Maciulaitis R., Janusonis T., Petrikaite V., Aukstakalniene A. (2006). Assessment of antibiotic use and comparison with recommendations for their rational use. Medicina (Kaunas).

[28] Al-Azzam S.I., Al-Husein B.A., Alzoubi F., Masadeh M.M., Al-Horani M.A.S. (2007). Self-medication with antibiotics in Jordanian population. Int. J. Occup. Med. Environ. Health.

[29] (2004). Raosoft Sample Size Calculator.

[30] Shehadeh M., Suaifan G., Darwish R.M., Wazaify M., Zaru L., Alja’fari S. (2012). Knowledge, attitudes and behavior regarding antibiotics use and misuse among adults in the community of Jordan: A pilot study. Saudi Pharm. J..

[31] Huang Y., Gu J., Zhang M., Ren Z., Yang W., Chen Y., Fu Y., Chen X., Cals J.W., Zhang F. (2013). Knowledge, attitude and practice of antibiotics: A questionnaire study among 2500 Chinese students. BMC Med. Educ..

[32] Dyar O.J., Pulcini C., Howard P., Nathwani D., ESGAP (ESCMID Study Group for Antibiotic Policies) (2014). European medical students: A first multicentre study of knowledge, attitudes and perceptions of antibiotic prescribing and antibiotic resistance. J. Antimicrob. Chemother..

[33] Dyar O.J., Howard P., Nathwani D., Pulcini C., ESGAP (the ESCMID European Society of Clinical Microbiology, Infectious Diseases Study Group for Antibiotic Policies) (2013). Knowledge, attitudes, and beliefs of French medical students about antibiotic prescribing and resistance. Med. Mal. Infect..

[34] Abbo L.M., Cosgrove S.E., Pottinger P.S., Pereyra M., Sinkowitz-Cochran R., Srinivasan A., Webb D.J., Hooton T.M. (2013). Medical students’ perceptions and knowledge about antimicrobial stewardship: How are we educating our future prescribers?. Clin. Infect. Dis..

[35] McNulty C.A., Cookson B.D., Lewis M.A.O. (2012). Education of healthcare professionals and the public. J. Antimicrob. Chemother..

[36] Azevedo M.M., Pinheiro C., Yaphe J., Baltazar F. (2013). Assessing the impact of a school intervention to promote students’ knowledge and practices on correct antibiotic use. Int. J. Environ. Res. Public Health.

[37] McNulty C.A., Nichols T., Boyle P.J., Woodhead M., Davey P. (2010). The English antibiotic awareness campaigns: Did they change the public’s knowledge of and attitudes to antibiotic use?. J. Antimicrob. Chemother..

[38] Widayati A., Suryawati S., de Crespigny C., Hiller J.E. (2011). Self medication with antibiotics in Yogyakarta City Indonesia: A cross sectional population-based survey. BMC Res. Notes.

[39] Ramalhinho I., Cordeiro C., Cavaco A., Cabrita J. (2014). Assessing determinants of self-medication with antibiotics among Portuguese people in the Algarve Region. Int. J. Clin. Pharm..

[40] Adriaenssens N., Coenen S., Versporten A., Muller A., Minalu G., Faes C., Vankerckhoven V., Aerts M., Hens N., Molenberghs G., Goossens H., ESAC Project Group (2011). European Surveillance of Antimicrobial Consumption (ESAC): Outpatient antibiotic use in Europe (1997–2009). J. Antimicrob. Chemother..

[41] Fincham J.E. (2008). Response rates and responsiveness for surveys, standards, and the journal. Amer. J. Pharm. Educ..

